# Correction: Triple-negative breast cancer suppressive activities, antioxidants and pharmacophore model of new acylated rhamnopyranoses from *Premna odorata*

**DOI:** 10.1039/d0ra90141e

**Published:** 2021-01-22

**Authors:** Abeer H. Elmaidomy, Rabab Mohammed, Asmaa I. Owis, Mona H. Hetta, Asmaa M. AboulMagd, Abu Bakar Siddique, Usama Ramadan Abdelmohsen, Mostafa E. Rateb, Khalid A. El Sayed, Hossam M. Hassan

**Affiliations:** Department of Pharmacognosy, Faculty of Pharmacy, Beni-Suef University Beni-Suef 62514 Egypt hossam.abdelazeem@pharm.bsu.edu.eg; Department of Pharmacognosy, Faculty of Pharmacy, Fayoum University Fayoum 63514 Egypt; Department of Pharmaceutical Chemistry, Faculty of Pharmacy, Nahda University Beni-Suef 62514 Egypt; School of Basic Pharmaceutical and Toxicological Sciences, College of Pharmacy, University of Louisiana at Monroe Monroe LA 71201 USA; Department of Pharmacognosy, Faculty of Pharmacy, Minia University Minia 61519 Egypt; Department of Pharmacognosy, Faculty of Pharmacy, Deraya University Minia 61111 Egypt; School of Computing, Engineering and Physical Sciences, University of the West of Scotland PA1 2BE Paisley UK

## Abstract

Correction for ‘Triple-negative breast cancer suppressive activities, antioxidants and pharmacophore model of new acylated rhamnopyranoses from *Premna odorata*’ by Abeer H. Elmaidomy *et al.*, *RSC Adv.*, 2020, **10**, 10584–10598. DOI: 10.1039/D0RA01697G

The authors regret that the name of the author Asmaa I. Owis was incorrectly spelled as Asmsaa I. Owis in the original article. The correct author list is as displayed in this document.

The Royal Society of Chemistry apologises for these errors and any consequent inconvenience to authors and readers.

## Supplementary Material

